# Molecular detection of *Anaplasma platys* in Pshdar Kurdish shepherd dogs of Sulaymaniyah Province, Iraq

**DOI:** 10.14202/vetworld.2024.2797-2801

**Published:** 2024-12-14

**Authors:** Eman D. Arif, Karwan M. Hama Khan, Israa H. Abd Al-Sada, Yousif M. S. Al-Barzinji

**Affiliations:** 1 Department of Microbiology, College of Veterinary Medicine, University of Sulaimani, Sulaymaniyah, Iraq; 2 Department of Animal Science, College of Agricultural Engineering Science, University of Sulaimani, Sulaymaniyah, Iraq; 3 Department of Anatomy and Histopathology, College of Veterinary Medicine, University of Sulaimani, Sulaymaniyah, Iraq; 4 Department of Animal Resources, College of Agricultural Engineering Science, Salahaddin University-Erbil, Iraq

**Keywords:** 16S ribosomal RNA gene, *Anaplasma platys*, blood, dogs, polymerase chain reaction

## Abstract

**Background and Aim:**

*Anaplasma platys* is a dog pathogen that causes anaplasmosis in various hosts, including humans. It is a rickettsial pathogen that causes cyclic thrombocytopenia in primary canine recipients and is spread by the brown dog tick *Rhipicephalus sanguineus*. This study aimed to map the genetic sequences of *Anaplasma* spp. isolates comparable with those of different global locations and determine the infection status of Pshdar Kurdish shepherd dogs from three regions in Sulaymaniyah province who did not exhibit clinical indications for *Anaplasma*.

**Materials and Methods:**

A total of 75 dog blood samples were collected from the center of the Sulaymaniyah, Dukan, and Ranya districts in the Sulaymaniyah province and subjected to polymerase chain reaction to determine the 16S ribosomal RNA (*16S rRNA*) gene of *A. platys*.

**Results:**

Only two dogs (2.7%) were positive for *A. platys*. The *16S rRNA* gene of *A. platys* was sequenced and registered in the National Center for Biotechnology Information GenBank with accession number OR467538. With four nucleotide changes, the sequence exhibited 99.72% similarity to strains identified as human infections and those found in recognized tick vectors.

**Conclusion:**

We conclude that the blood of Pshdar Kurdish shepherd dogs in the Sulaymaniyah region of Iraq contains a small number of *A. platys*. Moreover, the phylogenetic tree of the isolated species, *A. platys*, was significantly similar to other strains of *A. platys* found worldwide. In the Kurdistan region of Iraq, this study represents the first molecular detection of the *16S rRNA* gene of *A. platys*.

## Introduction

Numerous obligatory systemic bacterial infections that destroy the physiological health of humans and mammals belong to the *Anaplasmataceae* family. They are tiny, pleomorphic, coccoid, or ellipsoidal cells with a diameter of 0.3–0.4 m. They are discovered as inclusion bodies (morulae) in the cytoplasm of mammalian host cells. This family includes several recently emerging vector-borne agents with various life cycles involving mammalian and insect hosts [1]. Several species with a legitimate taxonomic status comprise the genus; according to Atif [2], *Anaplasma* comprises *Anaplasma marginale*, *Anaplasma centrale*, *Anaplasma bovis*, *Anaplasma ovis*, *Anaplasma caudatum*, and *Anaplasma phagocytophilum*. Cattle can contract infections from a variety of *Anaplasma* species, such as *A. bovis*, *A. phagocytophilum*, *A. centrale*, and *A. marginale*. *A. marginale* is known to be extremely harmful in bovines, but *A. centrale* serves as an antisense agent against anaplasmosis and is less virulent. Animals exposed to stress, heat, deworming, and movement develop severe sickness due to *A. ovis*. It creates an adequate threat to sheep, goat, and wild ruminants [3].

Two zoonotic illnesses that primarily affect dogs and wild canids are granulocytic anaplasmosis and infectious canine cyclic thrombocytopenia, which are caused by *Anaplasma platys* and *A. phagocytophilum*, respectively [4, 5]. *A. platys* was initially identified in 1978 as a cause of cyclic thrombocytopenia in dogs [6, 7]. Canine *A. platys* infections have various symptoms, including anorexia, lymph adenomegaly, splenomegaly, depression, weight loss, and thrombocytopenia [8, 9]. Most likely, the brown dog tick, *Rhipicephalus sanguineus*, is the biological vector of this pathogen [10, 11]. Dogs are frequently infected with *A. platys*, although cats, camels, and humans are also affected [12]. Compared with serology, which depends on antibodies that frequently exhibit interspecific cross-reactivity, polymerase chain reaction (PCR)-based diagnosis can be significantly more specific and offer data on the existing contamination grade [13, 14]. One of the shortcomings of these approaches is their dependence on the presence of pathogens in the bloodstream at the time of specimen collection [15]. A phylogenetic tree of *A. platys* strains employing genes from the blood of Pshdar Kurdish shepherd dogs in the Sulaymaniyah region, Iraq, has not been fully elucidated. A previous study in Iraq [16] aimed to recognize *A. platys* in dogs by ordinary PCR; however, the study only examined a variety of dog breeds from the Baghdad area.

This study aimed to investigate the infection status of Pshdar Kurdish shepherd dogs with no obvious clinical indicators of *A. platys* in the three regions of Sulaymaniyah province. In addition, a map of sequence similarities between *A. platys* isolates and those from other parts of the world was established.

## Materials and Methods

### Ethical approval

The study was approved by the Ethics Committee of College of Veterinary Medicine, Sulaimani University (Approval No. 01355/October 24, 2023).

### Study period and location

The study was conducted from November 2023 to May 2024 in the center of the Sulaymaniyah, Dukan, and Ranya districts in the Sulaymaniyah province.

### Animals and sample collection

In Iraq, Pshdar dogs are well-known shepherd guardians and watchdogs. The most well-known native dog in Kurdistan is considered a large breed of the tallest and most ferocious shepherd dog. In addition, the local Kurd population uses them for dogfight betting. In the Iraqi Kurdistan region, it is estimated that the native dog population does not exceed a few thousand dogs. The huge head of the Pshdar Kurdish dog is prominently convex, and it tends to conceal its length. Males with expanded bodies weigh more [17, 18]. In view of the lack of data on *A. platys* in dogs in the study area, we used the simple random sampling method. Apparently, healthy dogs were included in the study. Pshdar Kurdish dogs were employed to obtain 75 blood samples; 47 were obtained from the center of the Sulaymaniyah, 17 from Dukan, and 11 from Ranya. Whole blood samples were collected in ethylenediaminetetraacetic acid (EDTA) tubes (QeakLab, China) from the vena saphena magna and vena cephalica of Pshdar Kurdish shepherd dogs. To isolate and identify *Anaplasma*, the labeled test samples were placed in sterile plastic containers and transported in cool boxes (4°C) to the laboratory of the College of Veterinary Medicine at Sulaimani University.

### DNA extraction

We extracted genomic DNA from all 75 dog blood samples using the Quick-Gdna Mini Prep kit DNA (Zymo, USA) as per the manufacturer’s instructions. The genomic DNA concentration was measured using a Nanodrop spectrophotometer (Thermo, USA). We also confirm that the DNA was pure (absorbance at 260/280 nm).

### PCR amplification

The 451-bp segment sequences of the *16S ribosomal RNA* (*16S rRNA*) gene of *A. platys* were amplified using a set of oligonucleotide primers, forward PER1:5´-TTTATCGCTATTAGATGAGCCTATG -3´, and reverse PER2: 5´-CTCTACACTAGGAATTCCGCTAT -3´, as previously reported by Nasr *et al*. [19]. The whole DNA was amplified using the PCR GoTaq^®^ Green master mix kit (Promega, USA). The PCR tube was filled with 10 μL of the master mix, 5 μL of DNA, and 1 μL (10 pmol) of each forward and reverse primer. A final volume of 20 μL was achieved by adding 3 μL of diethylpyrocarbonate (DEPC) treated water. The thermal cycler technique (Techne Prime, UK) was used for the initial denaturation step, which lasted for 5 min at 95°C. After that, thermal cycling started with 40 cycles of denaturation (30 s at 95°C), annealing (for 30 s at 55°C), extension (for 30 s at 72°C), and final extension at 72°C for 5 min. After loading 7 μL onto a 1% agarose gel in 1× Tris/Borate/EDTA solution (Promega), the PCR products were separated. The gel was stained using a 5× safe dye (Clever, UK). Electrophoresis was performed using the Safe-Blue Illuminator/Electrophoresis System (Ingenius 3, UK) for 50 min at 120 V. Migration patterns were examined by comparing PCR product amplicons with a 100-bp DNA ladder (Gen Direx, Taiwan).

### Sequencing of *16S rRNA* gene

Thirty percent of PCR products were sequenced in both directions using a 3100 ABI PRISM sequencer (Applied Bio-systems, USA) with the above mentioned primers The obtained sequences were analyzed for similarity using Clustal Omega (https://www.ebi.ac.uk/jdispatcher/msa/clustalo), and single-nucleotide polymorphisms were identified. A phylogeny tree was constructed using the neighbor-joining method usingMolecular Evolutionary Genetics Analysis (MEGA X Software) (https://www.megasoftware.net/) [20].

## Results

### Identification of *16S rRNA*

**Figure-1:**
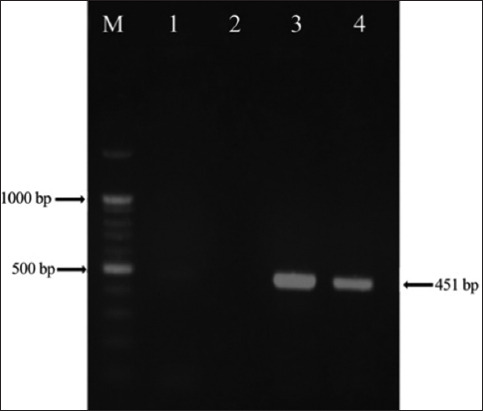
Polymerase chain reaction (PCR) amplifies the target DNA from *Anaplasma*. Lane M: DNA ladder (100 bp); Lanes 1, 3, and 4: *16S ribosomal RNA* gene of *Anaplasma* (451 bp); Lane 2: negative control (a PCR reaction mix free of DNA).

Using *16S rRNA*-PCR, all blood samples (75 dogs) from Sulaymaniyah, Dukan, and Ranya were examined for DNA from *A. platys*. The sample was positive if a 451-bp fragment was observed on a 1% agarose gel (Figure-1). The average anaplasma infection rate in dogs was 2.7% (2/75). *A. platys* was not found in the dog samples collected in the Ranya area (Table-1).

**Table-1:** Polymerase chain reaction results for *Anaplasma platys* in blood samples from Pshdar Kurdish dogs.

District name	Samples tested	Samples positive (N, %)
Sulaymaniyah	47	1 (2.1)
Dukan	17	1 (5.9)
Ranya	11	0.0
Total	75	2 (2.7)

### Sequence analysis

**Figure-2:**
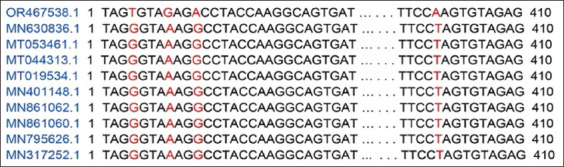
The multiple sequence alignment of *Anaplasma platys 16S ribosomal RNA* gene (OR467538) with related sequences downloaded from the National Center for Biotechnology Information.

**Figure-3:**
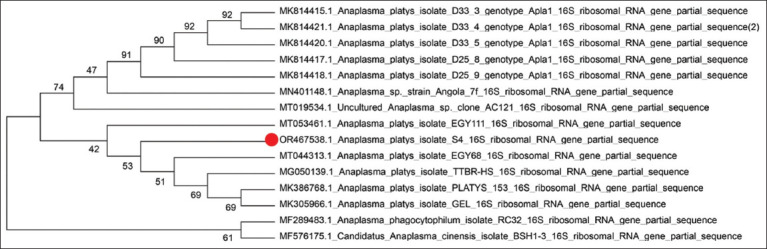
A neighbor-joining tree based on *Anaplasma platys* partial (451bp) 16S ribosomal RNA sequence (OR467538.1) and a few related strains available in GenBank. Bootstrap values are based on 1000 replicates.

The *16S rRNA* gene sequence of *A. platys* obtained in this study was submitted to the National Center for Biotechnology Information (NCBI) GenBank (accession number OR467538). The Basic Local Alignment Search Tool (BLAST) (https://blast.ncbi.nlm.nih.gov/Blast.cgi) search revealed that the present sequence had 99.72% similarity with the *16S rRNA* sequences of *A. platys* (MN630836.1, MT053461.1, MT044313.1, MT019534.1, MN401148.1, MN861062.1, MN861060.1, MN795626.1, and MN317252.1). Multiple sequence alignment indicated that the sequence obtained in the present study had four-point mutations at the nucleotides T.217 (G>T), A.221 (G>A), A.224 (G>A), and A.614 (T>A) in comparison with the sequences of *A. platys* mentioned above (Figure-2). With a sample of GenBank sequences, phylogenetic analysis illustrates that the present sequence is closest to the *16S rRNA* sequence of *A. platys* isolated from *R. sanguineus* ticks infested on domestic dogs in Egypt (MT053461.1, MT044313.1) (Figure-3).

## Discussion

This study is the first to identify detailed molecular evidence for *A. platys* organisms actively circulating in the blood of Pshdar Kurdish shepherd dogs without observable clinical signs in the Sulaymaniyah area. Some of the most common and fatal illnesses in dogs are caused by bacteria that are spread by feeding on blood arthropods like ticks [21]. Particularly significant and more prevalent in the Sulaymaniyah region is the tick, *R. sanguineus*, which can spread various vector-borne diseases, including *A. platys* [22]. Dogs with high levels of *Anaplasma* infections in circulation may be significant reservoir hosts, facilitating the steady spread of the disease among dogs, other vertebrates, and ticks. Dogs are the closest animals to humans, regardless of whether they are free-roaming dogs owned by people; hence, the risk of humans contracting *A. platys* is increased. Therefore, dogs may serve as sentinel species for the environmental monitoring and public health surveillance of potential human diseases [23].

In the present investigation, PCR, a rapid, highly sensitive, and accurate technology for detecting *A. platys*, was used on a large number of blood samples collected from Pshdar Kurdish shepherd dogs. In the present study, *A. platys* was detected in the observed models at a molecular frequency of 2.7%. This low-frequency rate is unpredictable because, given the positive environment, one would expect Sulaymaniyah to be an extremely widespread site for *A. platys*. Additional research is required to identify the reason for low detectable rates. However, subclinical and chronic anaplasmosis infections are more challenging to diagnose than acute infections. Therefore, to address this limitation, the use of PCR techniques should be considered [24]. In addition, the small number of positive dogs detected may have been affected by the fact that PCR sensitivity differs between laboratories.

The overall occurrence of dog anaplasmosis determined by this study was similar to that conducted in altered sections of the world, including the analysis that occurred in Colombia, where the infection rate using PCR was 2.2% (2/91) in sampled dogs for *A. platys* [25]. For *A. platys*, our result ratio was higher than those reported in the Philippines 0.6% (1/36) [26] and Qatar 1.6% (1/64) [27]. In comparison with two other studies from Thailand [28] and Palestine [24], they discovered markedly higher rates of infection of 12% (12/100) and 10% (13/135), respectively. In addition, the results of a previous study in pet dogs from Baghdad city using PCR showed a high infection rate (8%); *A. phagocytophilum* was found in 4.66% (7/150) of the cases, whereas *A. platys* was found in 3.33% (5/150) of the cases [16]. The scattering of vectors, socioeconomic variables, infection rank, modifications in weather and environmentalism, and the tests used to evaluate infection, as nested PCR or real-time PCR, all can contribute to variations in anaplasmosis prevalence worldwide [29].

Given the striking degree of similarity between the sequences reported within those and the literature obtained across this investigation, it is assumed that the introduction of animals from both surrounding and non-neighboring areas of Iraq might be the cause of anaplasmosis in dogs. Moreover, all of these isolates may have similar genealogical species relationships, according to the phylogenetic analysis of *Anaplasma* spp. using the *16S rRNA* gene, these findings are consistent with those of Cardoso *et al*. [30] and Koh *et al*. [31].

## Conclusion

The study findings show a low rate of *A. platys* in the blood of Pshdar Kurdish shepherd dogs in the Sulaymaniyah region of Iraq. The sequence similarity index and phylogenetic analysis revealed that this *Anaplasma* sequence was closest to the *16S rRNA* sequence of *A. platys*, which was previously reported. We recommend that subsequent studies should focus on collecting parasitic ticks from dogs or collecting blood samples from ticks to confirm their role in transmitting this pathogen.

## Authors’ Contributions

EDA: Designed the study. EDA and KMHK: Drafted the manuscript and data analysis. IHAA: Communicated with owners of dogs and collected samples. IHAA, YMSA, KMHK, and EDA: Laboratory investigations. All authors have read and approved the final manuscript.
